# Bone Turnover Markers in Response to Ultrasound-Guided Microwave Ablation for Primary Hyperparathyroidism

**DOI:** 10.3389/fendo.2021.782050

**Published:** 2021-12-01

**Authors:** Wenjing Ni, Yue Yuan, Xiaoqiu Chu, Guofang Chen, Xue Han, Jie Li, Xinping Wu, Jianhua Wang, Chao Liu, Shuhang Xu

**Affiliations:** ^1^ Endocrine and Diabetes Center, Affiliated Hospital of Integrated Traditional Chinese and Western Medicine, Nanjing University of Chinese Medicine, Nanjing, China; ^2^ Key Laboratory of TCM Syndrome & Treatment of Yingbing of State Administration of Traditional Chinese Medicine, Jiangsu Province Academy of Traditional Chinese Medicine, Nanjing, China; ^3^ Department of Ultrasound, Affiliated Hospital of Integrated Traditional Chinese and Western Medicine, Nanjing University of Chinese Medicine, Nanjing, China; ^4^ Department of General Surgery, Affiliated Hospital of Integrated Traditional Chinese and Western Medicine, Nanjing University of Chinese Medicine, Nanjing, China

**Keywords:** primary hyperparathyroidism, microwave ablation, thermal ablation, bone turnover, renal function

## Abstract

**Objective:**

To assess the efficacy and safety of ultrasound-guided microwave ablation (MWA) in the treatment of primary hyperparathyroidism (PHPT), and to investigate whether MWA can improve the bone turnover and renal function.

**Methods:**

A total of 20 consecutive PHPT patients with 21 parathyroid lesions treated with MWA in our center from May 2019 to March 2021 were recruited in this study. Serum parathyroid hormone (PTH), calcium and phosphorus levels before MWA and at 20 minutes, 4 hours, 1 day, 3 months, 6 months and 12 months after MWA were measured. Bone turnover biomarkers, renal function and lesion volume with volume reduction rate (VRR) before MWA and at the last follow-up were compared. Any complication related with MWA was evaluated. The technical and clinical success rates of MWA in the treatment of PHPT were calculated. Clinical success was defined as normal serum PTH and calcium without PHPT-associated manifestations at more than 6 months after ablation. Technical success was defined as complete ablation indicated by immediate postoperative contrast-enhanced ultrasound.

**Results:**

The serum PTH, calcium and phosphorus levels at their respective follow-up time points dropped significantly after MWA (*P <*0.05). The volume of parathyroid lesions at the final examination was significantly reduced, compared with pre-ablation volume (*P <*0.001), with a median VRR reaching 89%. The technical and clinical success rates were 100% and 63.6%, respectively. Substantial changes of bone turnover biomarkers were observed before and after MWA (*P <*0.05), but the differences in renal function were not statistically significant. No major complications were reported in all cases. Pre-MWA serum PTH, lesion volume, maximum diameter of lesion and ablation time were significantly different between patients with successful and failed MWA.

**Conclusions:**

PHPT can be effectively and safely treated by ultrasound-guided MWA, as proven by drop in serum PTH and reduction in the volume of parathyroid adenomas. Besides, MWA can impede bone remodeling to suppress hyperparathyroidism in the condition of PHPT.

## Introduction

Primary hyperparathyroidism (PHPT) is an endocrine disease caused by excessive secretion of parathyroid hormone (PTH) from one or more of the four parathyroid glands, manifesting elevated or normal serum calcium levels. Classical PHPT presents with a symptomatic, multiple system dysfunction, especially skeletal and renal damages. However, the clinical profile of PHPT has gradually changed from symptomatic to asymptomatic, due to multichannel examinations (1). Routine blood tests for serum PTH and calcium, frequent neck ultrasonography, as well as various imaging methods can by employed to identify asymptomatic PHPT (2). In China, the incidence of asymptomatic PHPT has increased from <21% in 2000-2006 to 42.4% to 52.5% in 2007-2010 (3).

Conventionally, parathyroidectomy (PTX) is the only definitive treatment for PHPT. Patients who do not meet the surgical indications or refuse surgery can choose medical treatment or just be observed. Nevertheless, PTX may bring about more blood loss and longer surgical time (4). Medications often present a limited clinical efficacy. As one of the commonly used calcimimetics, cinacalcet is unable to significantly improve bone mineral density (BMD) and urinary calcium excretion, although it does lower serum calcium level (5). Moreover, it often induces adverse events like headache, nausea and vomiting. Observations may be a good choice for asymptomatic patients with stable condition, but previous studies have shown that kidney stones and/or nephrocalcinosis present in 21% to 55% of patients with asymptomatic PHPT (6, 7). PHPT patients had higher a risk of fracture compared with healthy controls, especially in the forearm and spine sites. The risks of vertebral fractures were more than four times higher in PHPT patients than in those without skeletal and renal damages (8). Therefore, an early intervention of PHPT, even in mild condition (serum calcium <12mg/dL and associated with nonspecific symptoms), is still commonly required (5).

In the past few years, microwave ablation (MWA) has been considered as an alternative for PHPT patients, and its efficacy and safety have been initially clarified. Through literature review on PubMed, a total of 427 PHPT cases treated with thermal ablation have been reported since 2011, including 248 cases treated with MWA. The total success rate of thermal ablation to treat PHPT was 69.1% (**Table 1**). However, these studies did not assess the skeletal and renal involvement in PHPT patients before and after MWA. In the present study, we aimed to investigate the changes in PTH, serum calcium and volume of ablated lesions in PHPT patients, as well as evaluate skeletal and renal functions to further evaluate the efficacy and safety of ultrasound-guided MWA in the treatment of PHPT.

**Table 1:** Summary of thermal ablation studies in the treatment of PHPT from 2011~2021.

Year	Author	Thermal ablation type	Mean kilojoule or power	Ablation time	Follow-up time	Success rate (success number/total treatment number)
2011	Ambrosini (9)	HIFU	26.35KJ	Mean 40 min	12m	50.0 (2/4)
2012	Andrioli (10)	LA	2.067 ± 1.440KJ	–	Mean 54 ± 34m	0.0 (0/6)
2014	Kovatcheva (11)	HIFU	15.2 ± 7.7KJ	–	12m	23.0 (3/13)
2015	Jiang (12)	LA	1.739 ± 0.394KJ	9.66 ± 2.19 min	Mean 13.19 ± 0.98m	81.0 (17/81)
2016	Liu (13)	MWA	40W	26.75 ± 6.02 min	Mean 32.8 ± 17.9m	86.7 (13/15)
2018	Wolf (14)	RFA	9~40W	–	Immediately after ablation	55.6 (5/9)
2019	Fan (15)	MWA	35.68 ± 3.55W	129.14 ± 122.55 s	Mean 12.55 ± 5.21m	86.4 (19/22)
2019	Liu (4)	MWA	20~30W	22.0 ± 6.30 min	Mean 6m	82.1 (23/28)
2020	Wu (16)	MWA	35W	122.29 ± 107.54 s	24m	62.5 (5/8)
2020	Appelbaum (17)	LA	3.533 ± 1.893KJ	10.2 ± 1.2 min	24m	91.7 (11/12)
2020	Ha (18)	RFA	–	3.7 ± 3.4 min	13.6 ± 18.7m	63.6 (7/11)
2021	Wei (19)	MWA	30W	Median 170 s	Median 13.6m	89.4 (42/47)
2021	Wei (20)	MWA+RFA	30-40W	Median 129 s	Median 18.1m	89.9 (107/119)
2021	Erturk MS (21)	MWA	32.19 ± 4.20W	141.56 ± 23.98 s	6 m	87.5 (28/32)
2021	The present study	MWA	30W	Median 72.00 s	Median 6 m	63.6 (7/11)
Total						69.1 (289/418)

Case reports with 1~2 cases were not included. PHPT, primary hyperparathyroidism; MWA, microwave ablation; RFA, radiofrequency ablation; LA, laser ablation; HIFU, high-intensity focused ultrasound; m, month; s, seconds.

## Materials and Methods

### Patients

According to the inclusion and exclusion criteria, eligible patients with parathyroid adenoma-induced PHPT treated with MWA in the Affiliated Hospital of Integrated Traditional Chinese and Western Medicine, Nanjing University of Chinese Medicine from May 2019 to March 2021 were recruited in this study. Besides, another 9 healthy participants were included as controls in order to compare biochemical findings only and that procedure was not suitable for healthy subjects. They all met the following inclusion criteria for healthy controls: (i) serum PTH, calcium, phosphorus levels, bone turnover markers and renal function within normal ranges; (ii) no parathyroid lesions as well as no skeletal and renal involvements; (iii) no history of parathyroid diseases, fractures, nephrocalcinosis and kidney stones; (iv) no previous use of medications which interfere with bone metabolism and renal function; (v) no family history of parathyroid diseases. Fasting blood samples were all collected during 7:00 to 8:00 in the morning. Both serum PTH and biochemical bone markers were measured by electrochemiluminescence using the immunology analyzer (Cobas e602, Roche Diagnostics). Serum calcium, phosphorus, albumin, and creatinine were measured by colorimetry using the modular analyzer series (Cobas 702, Roche Diagnostics). This study was approved by the Ethics Committee of the Affiliated Hospital of Integrated Traditional Chinese and Western Medicine, Nanjing University of Chinese Medicine (2020SLKY005). Written informed consent was obtained from all subjects prior to the study.

Included were (i) symptomatic PHPT patients; (ii) asymptomatic PHPT patients who met one of the following criteria: serum calcium concentration more than 0.25 mmol/L above the upper limit of normal range, skeletal involvement or higher risk of fragility fractures indicated by biochemical parameters and/or imaging examination (T score of less than –2.5 at lumbar spine, hip or distal one-third radius, vertebral fracture by radiography, computed tomography (CT) or vertebral fracture assessment), or renal involvement (creatinine clearance less than 60 mL/min, nephrocalcinosis or kidney stone by ultrasound, CT or abdominal radiography); (iii) patients without history of neck surgery and signs of other malignant tumors; (iv) patients who were willing to receive MWA.

Excluded were patients showing (i) increase in serum PTH caused by thiazide diuretics, loop diuretics, lithium agents or bisphosphates; (ii) secondary and tertiary hyperparathyroidism; (iii) severe bleeding diathesis or coagulation disorders (prothrombin activity <60%, prothrombin time >18 seconds, or platelet count <60×10^9^/L); (iv) damage in vocal cords and recurrent laryngeal nerve examined by laryngoscopy; (v) cardiac insufficiency, hepatic failure or other severe organ dysfunctions; (vi) pregnancy and lactation.

### MWA Instrument and Techniques

MWA was performed using a microwave ablation system (KY-2000, Nanjing Canyon Medical Technology Co., Ltd.) producing 30 W of output power at a frequency of 2450 MHz. It contained an MWA instrument and an internally 16G water-cooled antenna for tumor ablation connecting with high efficiency detachable cable. The heat spot was 3 mm away from the tip of the ablation antenna. Siemens Acuson S2000 ultrasound machine was used for ultrasound-guided MWA with the 18L6 probe and the frequency of 6-18 MHz. SonoVue ultrasound contrast agent (pH 4.5-7.5) containing SF6 microbubbles with a mean diameter of 2.5 μm was gently mixed with 5 ml of normal saline, and 1.0 ± 0.2 ml of suspension was rapidly injected into the ulnar vein.

### MWA Procedures

To fully expose the neck area, the patient was placed in a supine position and monitored by electrocardiogram and pulse oximetry. The localization and extent of ablation areas, as well as the ablation path, were predetermined in ultrasound examinations. After disinfection using 0.2% iodoform and paving sterile sheets on the surgical area, ultrasound-guided local infiltration anesthesia was performed using 1% lidocaine. Through hydro-dissection technique, normal saline was continuously injected surrounding the parathyroid glands, to create a barrier to prevent thermal damage to the trachea, esophagus and recurrent laryngeal nerve. The ablation antenna was inserted into the lesion with the previously determined path, and MWA producing 30 W of output power was started until surrounding important organs and tissues were hydrodissected. Specifically, the ablation antenna was fixed into the central of small parathyroid lesions to initiate a fixed ablation, while in cases with large parathyroid lesions, its tip was adjusted until all the parathyroid lesions were covered by the transient hyperechoic zone. After the ablation, color Doppler ultrasound and contrast-enhanced ultrasound were performed. The defect in the parathyroid glands was observed by contrast-enhanced ultrasound, in which no enhancement indicated MWA-induced coagulative necrosis. The ablation antenna was gently pulled out and the ablation area was ice-packed for at least 4 h. Vital signs, phonation, swallowing and any other discomforts were closely monitored during and after MWA. MWA of each recruited patient was performed by the same operator with high-volume thermal ablations experience.

### Evaluation Criteria

#### Efficacy Indexes

Clinical success was defined as normal ranges of serum PTH and calcium without PHPT-associated manifestations at more than 6 months after ablation. A technical success was defined as complete ablation indicated by immediate postoperative contrast-enhanced ultrasound. Recurrent PHPT was defined as recurrence of hypercalcemia after a normocalcemic interval at more than 6 months after operation (22). The volume (V) of the parathyroid glands was calculated using the equation V (cm^3^) = πabc/6, where a, b and c were the three maximum perpendicular diameters shown on the ultrasound image. The volume reduction rate (VRR) of the parathyroid glands was calculated using the equation VRR (%) = ([initial volume–final volume] × 100%)/initial volume. Power of ablation per unit volume was calculated as $\frac{\text{ablation time}\times \text{power}}{\text{volume of lesion}}$ .

#### Safety Indexes

Major complications refer to permanent injuries leading to substantial morbidity and disability that increased care intensity, resulted in hospital admission, or substantially lengthened the hospital stay, such as permanent nerve injuries and permanent hypoparathyroidism (23). All other complications were considered minor (e.g., postoperative pain, bleeding, infection, swelling, hoarseness, choking when drinking and difficulty in swallowing).

### Follow-Up

Baseline characteristics were all recorded. No patients were treated with vitamin D before MWA. However, all patients received vitamin D supplementation after MWA to avoid numbness or tetany. Blood samples were intravenously collected before MWA and at 20 minutes, 4 hours, 1 day, 3 months, 6 months and 12 months after MWA for measuring serum PTH, calcium and phosphorus. Bone turnover and metabolic markers, including (25(OH)D, ALP, osteocalcin, total (P1NP), and β-CTX), and renal function (serum creatine and eGFR) were assessed before the ablation and the last follow-up. The volume as well as volume reduction rate of parathyroid lesions were compared before treatment and during the follow-up. MWA time, postoperative complications and their duration, treatment and outcomes were recorded.

### Statistical Analyses

SPSS 22.0 was used for statistical analyses. According to the normality of data distribution, parametric or nonparametric tests were applied. Normality of distribution was assessed using the Kolmogorov-Smirnov test. Continuous variables in normal distribution were presented as the mean ± standard deviation and analyzed with t Student test. Measurement data not in normal distribution were expressed as M (P25, P75), and compared by the Wilcoxon signed rank test. The Mann-Whitney U test was used for the comparison of data not in normal distribution. Both univariate and multivariate Cox regression analyses were performed to predict the potential parameters on the influence of treatment failure. *P <*0.05 was considered as statistically significant.

## Results

### Baseline Characteristics

A total of 20 consecutive patients with 21 parathyroid adenomas with a median age of 45.00 (31.75, 56.25) years were recruited in this study, involving 3 males and 17 females. Among the 18 asymptomatic patients, 11 patients had organ damage, mostly manifested by bone loss and osteoporosis. Only 2 patients had overt clinical symptoms. One female patient experienced persistently progressive ostalgia and the other female patient had intermittent legs twitching, emotional instability and memory less. Before ablation, the median volume and maximum diameter of 21 lesions were 0.71 (0.42, 3.04) cm^3^ and 1.87 (1.45, 2.50) cm, respectively. The median preoperative levels of serum PTH, serum calcium and phosphorus were 122.35 (91.52, 266.90)pg/mL, 2.72 ± 0.30 mmol/L and 0.85 ± 0.16 mmol/L, respectively. Specifically, bone metabolic profiles showed that 82.4% (14/17) patients had 25(OH)D insufficiency or deficiency, and abnormally higher levels of total P1NP and β-CTX accounted for 76.90% (10/13) and 84.60% (11/13) of patients, respectively. The median duration follow-up was 6 (3, 12) months (**Table 2**). In the control group, the median age of 9 patients (8 females; 1 male) was 38.00 (35.00, 46.50) years, which was comparable to PHPT patients. The median PTH, calcium and phosphorus levels were 39.76 (31.55, 57.24) pg/mL, 2.32 (2.24, 2.36) mmol/L and 1.08 (1.02, 1.23) mmol/L, respectively, all statistically different from those of PHPT patients before MWA (*P <*0.001).

**Table 2:** Baseline characteristics of patients with PHPT.

Characteristics	Data
**Sex**	
Male	3
Female	17
premenopausal female	10
postmenopausal female	7
**Mean age (years)**	45.45 ± 13.68
<50	13
≥50	7
**Symptoms related to PHPT**	
Yes	2
No	18
**Laboratory results**	
PTH (pg/mL)	122.35 (91.52, 266.90)
Calcium (mmol/L)	2.72 ± 0.30
Phosphorus (mmol/L)	0.85 ± 0.16
25(OH)D (ng/mL)	13.1 (10.2, 17.8)
ALP (U/L)	89 (73, 167)
Osteocalcin (ng/mL)	35 (21, 61)
Total P1PN (ng/mL)	89.46 (64.57, 158.60)
β-CTX (pg/mL)	1099.00 (731.90, 1391.00)
Creatinine (μmol/L)	67 (60, 74)
eGFR(mL/min/1.73 m^2^)	89.6 (76.5, 106.7)
**Nodules**	
Location of nodules	
superior left	2
inferior left	5
superior right	6
inferior right	8
Size of ablated nodules	
median volume (cm^3^)	0.71 (0.42, 3.04)
median value of maximum diameter (cm)	1.87 (1.45, 2.50)
**MWA**	
power (W)	30
median ablation time (s)	72.00 (55.00, 120.50)

PHPT, primary hyperparathyroidism; PTH, parathyroid hormone; 25(OH)D, 25-hydroxyvitamin D; ALP, alkaline phosphatase; P1PN, procollagen type 1 N-telopeptide; β-CTX, C-terminal telopeptide of β-I collagen; eGFR, estimated glomerular filtration rate.

### Overall Efficacy

The serum PTH levels at 20 minutes, 4 hours, 1 day, 1 month, 3 months, 6 months and 12 months after MWA were all significantly lower than those before MWA (*P <*0.05). Compared with those before MWA, the serum calcium levels also decreased significantly at 20 minutes, 4 hours, 1 day, 1 month, 3 months, 6 months and 12 months after MWA (*P <*0.05). The serum phosphorus levels were significantly higher at 20 minutes, 1 day, 1 month, 3 months and 12 months after MWA (*P <*0.05). The clinical success rate at 1 month, 3 months, 6 months and 12 months after ablation were 42.1% (8/19), 52.6% (10/19), 50.0% (5/10), 75.0% (6/8), respectively (**Table 3**). Taken together, the overall clinical success rate was 63.6%. All symptoms related to PHPT disappeared after treatment.

**Table 3:** Changes in serum PTH, calcium, phosphorus levels, volume and the maximum diameter of parathyroid lesions in PHPT patients before and after MWA.

Follow-up period	Serum PTH (pg/mL)	Serum calcium (mmol/L)	Serum phosphorus (mmol/L)	V (cm^3^)	Maximum diameter (cm)
Before MWA (n=20)	122.35 (91.52, 266.90)	2.72 ± 0.30	0.85 ± 0.16	0.71 (0.42, 3.04)	1.87 (1.45, 2.50)
20 min after MWA (n=20)	48.35 (19.99, 93.82)^***^	2.64 ± 0.28	0.78 ± 0.16^*^	–	–
4 h after MWA (n=20)	35.36 (12.98, 76.32)^***^	2.61 ± 0.21^*^	0.76 ± 0.29	–	–
1 day after MWA (n=20)	48.10 (21.97, 67.00)^***^	2.35 ± 0.19^***^	1.05 ± 0.29^**^	–	–
1 month after MWA (n=19)	72.14 (44.57, 104.20)^**^	2.35 ± 0.10^***^	1.03 ± 0.18^**^	0.55 (0.30, 0.86)^**^	1.25 (1.00, 1.70)^**^
3 months after MWA (n=19)	68.30 (58.96, 101.00)^**^	2.37 ± 0.19^**^	1.00 ± 0.14^**^	0.26 (0.07, 0.40)^**^	1.08 (0.74, 1.65)^**^
6 months after MWA (n=11)	67.69 (42.24, 82.26)^**^	2.38 ± 0.11^*^	0.95 ± 0.15	0.10 (0.03, 0.30)^**^	0.86 (0.45, 1.34)^*^
12months after MWA (n=8)	59.61 (39.17, 66.95)^*^	2.37 ± 0.15^**^	1.06 ± 0.23^*^	0.04 (0.00, 0.16)^**^	0.50 (0.00, 1.10)^*^

PTH, parathyroid hormone; PHPT, primary hyperparathyroidism; MWA, microwave ablation; V, volume of parathyroid glands; -, not reported; n means patient number.

Compared with preoperative levels, *P < 0.05, **P < 0.01, ***P < 0.001.

Normal reference ranges: serum PTH 15-65 pg/mL, serum calcium 2.10-2.55 mmol/L, serum phosphorus 0.81-1.45 mmol/.

**Figure 1:**
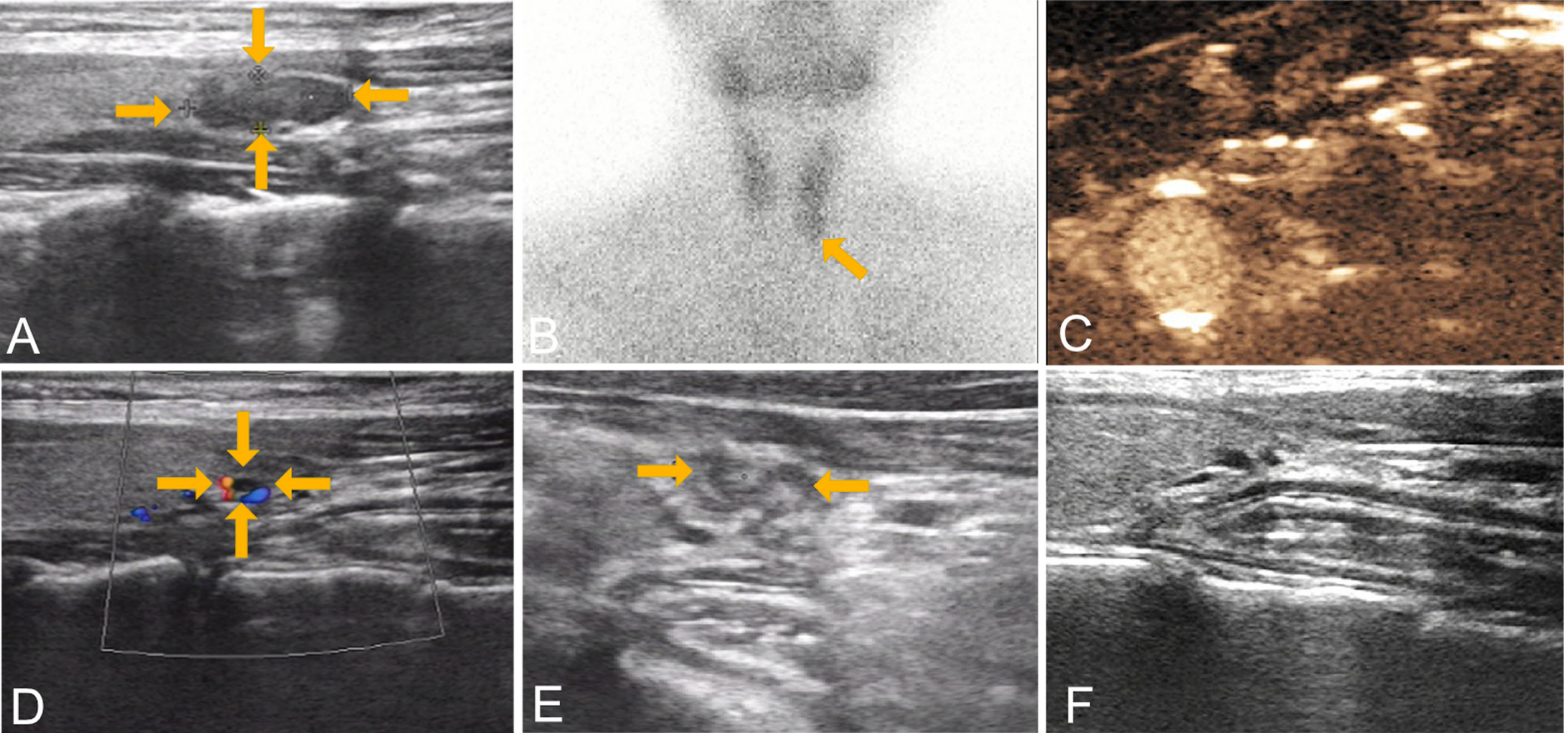
Images from a 30-year-old female with a left lower parathyroid adenoma (13.0 mm × 4.4 mm × 1.3 mm) who underwent microwave ablation therapy. **(A)** preoperative gray scale ultrasound; **(B)** preoperative radionuclide scanning; **(C)** immediate contrast-enhanced ultrasound after ablation; ultrasound examination at three months **(D)**, six months **(E)** and twelve months **(F)**. Shown by ultrasound, the volume reduction rates were 92%, 98% and 100%, respectively. PHPT, primary hyperparathyroidism; MWA, microwave ablation.

The median ablation time was 72.00 (55.00, 120.50) seconds. Contrast-enhanced ultrasound after MWA showed that all patients underwent complete ablation. The technical success rate was 100%. The volume and maximum diameter of parathyroid lesions after MWA were significantly reduced, compared with those before ablation during the follow-up (*P <*0.05) (**Table 3**). The median VRR reached 89%, with 17 lesions reduced by more than 50% at the last follow-up. The maximum volume and diameter of parathyroid glands measured before MWA were 4.08 cm^3^ and 2.70 cm, then reduced to 0.04 cm^3^ and 0.66 cm at 12 months, respectively. Noteworthy, as shown on follow-up ultrasound images, the volume of ablated lesion shrank gradually over time, and became evidently smaller after three months of MWA. In this study, parathyroid lesions completely disappeared on ultrasonography in 3 patients at the last follow-up (**Figure 1**).

We divided the patients followed up by at least 6 months into ablation success and failure group, and further investigated the factors related to ablation failure. There were significant differences in serum PTH, lesion volume, maximum diameter of lesion before MWA, and ablation time between the two groups (**Table 5**).

### Effects on Bone Metabolic Profiles and Renal Function

Changes in bone turnover markers were observed before and after MWA (**Table 4**). Both bone formation (ALP, osteocalcin, total P1NP) and resorption (β-CTX) markers decreased significantly after treatment, by 24%, 40%, 41%, 63% for ALP, osteocalcin, total P1NP and β-CTX, respectively. Notably, β-CTX normalization rates achieved 100% (13/13) at the last follow-up. Besides, the median concentration of 25(OH)D at the last follow up was significantly higher compared with that before MWA (*P <*0.01). There were significant differences in osteocalcin, total P1NP and β-CTX levels after ablation between clinical success group and failure group (all *P <*0.05), but the differences in 25(OH)D and ALP levels after ablation were not statistically significant. Further analysis showed that post-MWA PTH was associated with post-MWA osteocalcin, total P1NP and β-CTX levels (*P*=0.026, *P*=0.017 and *P*=0.026, respectively). However, post-MWA calcium level was not associated with post-MWA osteocalcin, total P1NP and β-CTX levels (all *P* >0.05). In terms of renal function, creatinine and eGFR levels rose slightly after MWA, but the differences were not statistically significant (**Table 4**).

**Table 4:** Differences in bone metabolic profiles and renal function between PHPT patients and controls.

	Patients Before MWA	Patients after MWA^a^	The control group
25(OH)D (ng/mL)	13.1 (10.2, 17.8)^*^	18.5 (15.6, 21.6)^△△^	17.7 (15.25, 25.15)
ALP (U/L)	89 (73, 167)^**^	72 (51, 100)^△△^	61 (49, 73)
Osteocalcin (ng/mL)	35 (21, 61)^***^	22 (14, 36)^*△△^	13 (10, 15)
Total P1PN (ng/mL)	89.46 (64.57, 158.60)^**^	61.85 (32.40, 89.37)^△△^	48.87 (27.96, 50.51)
β-CTX(pg/mL)	1099.00 (731.90, 1391.00)^***^	372.30 (197.15, 498.15)^△△^	310.60 (167.65, 447.05)
Creatinine (μmol/L)	67 (60, 74)^*^	69 (59, 75)^*^	54 (52, 67)
eGFR (mL/min/1.73 m^2^)	89.6 (76.5, 106.7)^**^	94.3 (74.6, 109.2)^*^	119.14 (103.07, 129.44)

MWA, microwave ablation; PHPT, primary hyperparathyroidism; PTH, parathyroid hormone; 25(OH)D, 25-hydroxyvitamin D; ALP, alkaline phosphatase; P1PN, procollagen type 1 N-telopeptide; β-CTX, C-terminal telopeptide of β-I collagen; eGFR, estimated glomerular filtration rate.

Compared with the control group, *P<0.05, **P<0.01, ***P<0.001; Compared with patients before MWA, ^△△^P < 0.01.

a The last follow-up results were listed in patients after MWA.

Serum 25(OH)D level <20 ng/mL, <12 ng/mL, between 20~30 ng/mL and ≥30 ng/mL are considered as vitamin D insufficiency, deficiency, moderate and sufficiency respectively.

Normal reference ranges: ALP 35~100 U/L. Osteocalcin 11~43 ng/mL women before menopause, 15~46 ng/mL women post menopause; 24~70 ng/mL, 14~42 ng/mL and 14~46 ng/mL for man between 18~30 years old, 30~50 years old and 50~70 years old, respectively; 13~48 ng/mL for patients with osteoporosis. P1NP 15.13~58.59 ng/mL, 16.27~73.87 ng/mL and 9.06~76.24 ng/mL for premenopausal women, postmenopausal women and men, respectively. β-CTX 25.00~573.00 pg/mL, 104.00~1008.00 pg/mL and 16.00~584.00 pg/mL for premenopausal women, postmenopausal women and men, respectively; Creatinine: 45~84 μmol/L; eGFR was calculated using Chinese modified modification of diet in renal disease (MDRD) study equation, eGFR(mL/min/1.73 m^2^)=186×creatinine^-1.154^×age^-0.203^×0.742 (female). The renal dysfunction was defined as eGFR<60 mL/min/1.73m^2^.

The intra-assay variation: PTH 2.1%; ALP 2.2%; 25(OH)D 6.7%; osteocalcin 1.3%; P1PN 1.7%; β-CTX 1.5%.

The inter-assay variations: PTH 1.4-1.7%; ALP 0.6-0.7%; 25(OH)D 2.7-4.6%; osteocalcin 0.9-1.3%; P1PN 1.2-1.6%; β-CTX 1.4-1.6%.

There were significant differences in bone turnover markers, creatinine and eGFR levels between the controls and PHPT patients treated before MWA. Between them, there were no significant differences in 25(OH)D, ALP, total P1NP and β-CTX levels, but osteocalcin and renal function indexes demonstrated statistical differences (**Table 5**).

**Table 5:** Comparisons between success group and failure group.

	Success group (n = 7)	Failure group (n = 4)
Age (y)	41.00 (28.00, 54.00)	48.50 (33.50, 65.00)
follow-up time (m)	12 (12, 12)	9 (6, 12)
Preoperative PTH (pg/mL)	98.09 (88.90, 221.4)^*^	437.10 (191.30, 566.70)
Preoperative calcium (mmol/L)	2.63 (2.28, 2.73)	2.80 (2.54, 3.12)
Preoperative phosphate (mmol/L)	0.89 (0.80, 0.97)	0.77 (0.55, 1.01)
Preoperative volume (cm^3^)	0.50 (0.39, 0.71)^*^	4.35 (1.87, 4.79)
Preoperative maximum of diameter (cm)	1.40 (1.26, 1.87)^**^	2.40 (2.10, 2.85)
Ablation time (s)	57.00 (40.00, 67.00)^*^	134. 50 (78.50, 205.50)
power of ablation per unit volume (s*w/cm^3^)	2748.57 (1669.25, 6556.91)	1234.13 (661.21, 2292.13)

Compared with the failure group, *P < 0.05, **P < 0.01.

### Complications

No serious life-threatening complications were reported during MWA and follow-ups. Mainly, the postoperative minor complications included transient mild pain, voice change or hoarseness, and numbness in the hands and feet. Three patients with hoarseness relieved within one week to one month. Only one patient had numbness of the whole body, choked drinking, and vomiting, whose serum calcium level (2.34 mmol/L) on the first day of MWA was in the normal range. After oral administration of 10 ml of 10% calcium gluconate and intramuscular injection of 5 mg vitamin D_2_, the above-mentioned symptoms were alleviated and not reported in the follow-up period. Taken together, all patients could tolerate MWA well.

## Discussion

Thermal ablations have been used to treat primary hyperparathyroidism for a decade, including high-intensity focused ultrasound (HIFU) (9, 11), laser ablation (LA) (10, 12, 17), radiofrequency ablation (RFA) (14, 18) and MWA (13, 15, 19, 21). Although their mechanisms to produce heat vary a lot, thermal ablation techniques produce heat to ablate lesions through a mechanism of tissue necrosis. Currently, MWA is the most widely used type of thermal ablation in PHPT treatment, and the cure rate was 80.0%~100.0%, which was comparable to surgery (4). However, few studies reported the therapeutic effect on target organs of bone and kidney. Only one study presented BMD improvement after 2 years’ MWA in PHPT patients (16). To our knowledge, this is the first study to evaluate the bone metabolic profiles and renal function of PHPT patients treated with MWA.

In the present study, the median level of 25(OH)D increased from 13.1 ng/mL to 18.5 ng/mL post ablation. Compared with healthy people, PHPT patients are more likely to complicate with vitamin D deficiency or insufficiency (5). Although most patients recruited in our study had a slight increase in 25(OH)D level, their vitamin D levels were not in the normal range. The American Association of Endocrine Surgeons recommended a short-term supplementation of calcium and/or vitamin D in postoperative PHPT patients, which may favor patients treated with MWA (22). In this study, all patients received vitamin D supplementation after ablation to avoid numbness or tetany. A meta-analysis reported that vitamin D supplementation in patients with PHPT and vitamin D deficiency could significantly reduce PTH levels without causing hypercalcemia and hypercalciuria (24), indicating it is an effective and safety treatment in PHPT condition.

The levels of bone turnover markers rise in PHPT, reflecting accelerated bone remodeling cycle. Bone resorption is always coupled with bone formation. Chronically increased PTH favors osteoclast to increase resorption, followed by the release of a number of growth factors from bone matrix to stimulate osteoblast migration, differentiation, and function (25). Hence, bone formation speeds up, resulting in rapid release of cell-derived bone turnover markers detected in the circulation (26). Although the effects of PTH on bones are still obscure, decreased BMD and increased bone turnover markers have been observed in PHPT patients. A study reported that MWA improved BMD in PHPT patients after 2 years’ follow-up (16). As bone turnover markers act as more sensitive indicators to reflect the fine changes in bone condition, it is meaningful to evaluate them **before and after MWA.**

We observed that the level of osteocalcin decreased significantly after ablation. Osteocalcin regulates the regulation of bone resorption, but also the mineralization of the matrix and differentiation of osteoblasts, a mechanism that maintains the normal mineralization of bone, inhibits the mineralization of cartilage and the abnormal hydroxyapatite crystal formation of bone. A study from Poland found that the osteocalcin levels of 17 PHPT patients returned to normal at six months after surgery (27). In our study, although osteocalcin did not fall into normal ranges in all patients, the median level of osteocalcin significantly decreased from 35 ng/mL to 22 ng/mL, indicating the improvement of bone turnover status. Another bone formation marker ALP also significantly dropped from 89 U/L before MWA to 72 U/L at the final examination. The lower bone formation rate after ablation was consistent with those in other studies on thermal ablation in the treatment of PHPT (19).

Serum P1NP, a specific and sensitive index of new bone formation, reflects the ability of osteoblasts to synthesize collagen, while β-CTX is a marker of bone resorption reflecting the bone resorption activity of osteoclasts. We observed total P1NP significantly decreased from 89.46 ng/mL to 61.85 ng/mL and β-CTX lowered from 1099.00 pg/mL to 372.30 pg/mL, which was comparable to the response after surgery (28). To be noted, the levels of β-CTX in 13 patients altogether fell into the reference intervals at the last follow-up. Costa AG et al. (29) found that the levels of bone resorption markers dropped most rapidly after surgical removal of parathyroid glands, providing a ‘‘window’’ when bone formation is accelerated due to the initial and preferential reduction in bone resorption after PTX. In patients treated with MWA, β-CTX is also a sensitive index to track the influence of hyperparathyroidism on bone.

Nine healthy subjects were included as the control group. There were no significant differences in 25(OH)D, ALP, total P1NP and β-CTX between PHPT patients after MWA and the control group, indicating that MWA can lower some certain bone turnover markers of PHPT patients to those of healthy controls. Abnormally high levels of bone turnover markers in PHPT patients indicate the potential bone loss and osteoporosis, which may further increase the risk of osteoporotic fractures. Furthermore, a retrospective study from Brazil demonstrated minimum preoperative values of 0.684 ng/mL for β-CTX and 76.0 ng/mL for P1NP in PHPT patients were associated with a ≥4% increase in BMD (30), making biochemical bone markers a surrogate tool to monitor the progression of PHPT.

Renal dysfunction due to or followed by nephrolithiasis, is a severe complication of PHPT. Approximately 12% (31) to 30.4% (32) PHPT patients are combined with impaired renal function (eGFR less than 60 mL/min/1.73 m^2^). Liang CC et al. (33) reported that PTX could slow the decline of eGFR, but another study did not find PTX could reverse renal dysfunction (32). Scarce data were available to evaluate the renal function of PHPT patients after treatment. In this study, marginal increase in creatinine and eGFR levels were observed, but the differences did not show statistical significance, perhaps due to our relatively small sample size, short follow-up duration and selection bias of retrospective study. Although the relationship between PHPT and renal function has not been clarified, chronic increase in PTH level may serve as a risk factor for kidney function impairment by accelerating endothelial injury and subsequent organ fibrosis (34). Further research is needed to assess the association of renal function with PHPT in patients treated with different options.

After 6-12 months of follow-up, two patients failed to show normal PTH or calcium levels. A multicenter study reported that the 7 out of 114 patients had recurrent PHPT during a follow-up period of 18.1 months (20). They found that a maximum diameter less than 0.6 cm resulted in missed ablation. In this study, we found that pre-MWA serum PTH, lesion volume, maximum diameter of lesion and ablation time were significantly different between the two groups, a finding that is consistent with those reported in other studies (35). Besides, another two patients had PTH slightly exceeding the upper limit of normal range. In PHPT treatment, normocalcemic parathormone elevation (NPE), characterized by a normal range of serum calcium with elevated PTH levels, has been detected in PHPT patients treated with PTX (36). During a short-term follow-up of 3-18 months and a long-term follow-up of 10 years after surgery, 11% (37) to 44% (38) of PHPT patients developed NPE, respectively. It is speculated that peripheral resistance to PTH may exist in PHPT patients, and adaptive compensation of serum PTH fails to restore calcium homeostasis after treatment of PHPT, leading to abnormal PTH after surgery (38). In addition, this abnormality can also be attributed to differences in baseline PTH, serum calcium level, vitamin D status, lesion volume (35, 39), follow-up durations (40) and clinical experiences. Interestingly, with the prolongation of observation after operation, the proportion of NPE decreased gradually, and NPE was not correlated with the recurrence of PHPT (37, 40). No major complications occurred during the process of MWA and follow-ups, verifying that MWA is a safe therapy for PHPT. In this study, three patients experienced with hoarseness, partly due to the heat produced by thermal ablation stimulating the recurrent laryngeal nerves. Therefore, the recurrent laryngeal nerves should be protected during thermal ablation.

A limitation of our study is a relatively low success rate compared with other studies of MWA, but this rate was parallel with that of HIFU (9). Nevertheless, from post-ablation 1 month to 12 months, the clinical success rate gradually raised. We supposed during a longer follow-up, a higher success rate may be observed. Shadowed by COVID-19 outbreak, the relatively short observation duration, incomplete data as well as small simple size potentially influenced the final success rate.

In conclusion, this study verified that MWA is a reasonable option in the treatment of PHPT, especially asymptomatic PHPT patients with potential target organ damage or a mild condition. We provided evidence that ultrasound-guided MWA effectively decreases levels of abnormal bone turnover markers and thus improve bone health of PHPT patients. In the future, perspective studies with large samples are required to identify the long-term efficacy and safety of thermal ablation for PHPT, and its long-term effects on bone health and renal function in PHPT patients.

## Data Availability Statement

The raw data supporting the conclusions of this article will be made available by the authors, without undue reservation.

## Ethics Statement

The studies involving human participants were reviewed and approved by Affiliated Hospital of Integrated Traditional Chinese and Western Medicine, Nanjing University of Chinese Medicine. The patients/participants provided their written informed consent to participate in this study. Written informed consent was obtained from the individual(s) for the publication of any potentially identifiable images or data included in this article.

## Author Contributions

WN and YY developed the research questionnaire and wrote the protocol for this study. WN, XH, and SX were responsible for the original study design and data collection together with the other authors. WN and YY analyzed the data. SX, JL, XW, and JW participated the diagnosis and treatment of PHPT. XC, GC, and SX interpreted the results. WN and YY wrote the article. CL and SX revised it critically for important intellectual content. All authors agreed to take responsibility for the integrity of the data and the accuracy of the data analysis. All authors have approved the final version of the manuscript.

## Funding

Design and data collection were supported by the grant supports of Jiangsu Provincial Key Research and Development Program (BE2020726) and the Medical Scientific Research Foundation of Jiangsu Province of China (Surface project) (M2020102).

## Conflict of Interest

The authors declare that the research was conducted in the absence of any commercial or financial relationships that could be construed as a potential conflict of interest.

## Publisher’s Note

All claims expressed in this article are solely those of the authors and do not necessarily represent those of their affiliated organizations, or those of the publisher, the editors and the reviewers. Any product that may be evaluated in this article, or claim that may be made by its manufacturer, is not guaranteed or endorsed by the publisher.
